# Hospital Housekeeping

**Published:** 1898-09-03

**Authors:** 


### HOSPITAL HOUSEKEEPING.

By a late Matron.

III.

I am fully convinced, after much experience and extended observation of the hospital systems of many foreign countries that the weakest spot in our hospital régime lies in the kitchen department. And it appears to me a comparatively simple matter to set our hospital kitchens, pantries, and larders in scientific order. And the first step is to place the cuisine of the institution on a more or less intellectual and trained platform.

Many matrons tell me that they have found a course at the Sanitary Institute to be of the greatest benefit to them in hospital housewifery, more especially in the food department. The instruction, both practical and theoretic, given at the Institute, as to the physical signs and evidences of adulteration in foods proves most valuable.

And we must admit that a hospital housekeeper should be able to detect palpable adulteration and inferiority in the quality of food, just as the trained nurse learns to note the signs of physical disease in her patients.

Whilst acknowledging the value of the Sanitary Institute instruction from the housewifery point of view, I can see that it is not altogether all that is needed for the education of the hospital housekeeper. Neither should I regard with favour the introduction to England of a class of professional or "institution buyers," such as exist in the United States. The system of "professional buying" is open to many objections. But it appears to me that we undoubtedly need in every hospital training school a detailed, perfected, and practical method of teaching hospital economics—this being more specially applied to the buying, the storing, and the preservation of food stuffs generally.

In the present article I am dealing with food as raw material, else I might have much to say anent the cooking departments of our hospitals. But that, as Kipling says, "is another story." To take one point alone, it has always appeared to me that the milk and butter supplies of our hospitals is a dietetic question which needs dealing with rather more carefully than is usually the case. The sick are much more susceptible than are the well to any additions to milk of boracic, salicylic, and other such commercial "preservatives." And for this reason such should not exist in milk and butter served to hospitals. I stand open to correction if I am wrong in my conclusions as to hospitals generally, but after a very extensive experience of internal hospital management, I cannot recall that any test analysis was ever employed towards the foods supplied to the hospitals in which I have been engaged. This may be done in some hospitals, but so far as my knowledge takes me I have not come across such an existent system.

Presumably the drugs provided for our hospitals are subject to expert test and analysis; and foods—the "kitchen medicines"—should certainly be periodically tested and examined. It surely is an anomaly that whilst urine testing is made a special feature in the training of the nurse, that the same nurse should be totally unable to test the purity and quality of the milk she administers to her patients, and that she should also be quite incompetent to judge of the condition and quality of the beef wherefrom she proposes to manufacture beef-tea for her sick dependents. That this should be so is a weak spot in the practicability of nurse-training—and practicability is what we most need in order to turn out the best kind of nurse. I quite agree with that sentiment of a recent writer in The Hospital, that it is doubtless a fine thing to talk learnedly of reflexes and anti-toxins, but that this kind of learning does not produce the best kind of nurse.

Sound practical common-sense knowledge of the cuisine, dietetics, and housewifery come "away ahead," as our Transatlantic friends would say. Let the anti-toxins and reflexes be added afterwards; they are the "accomplishments," the trimmings added to the solid good wearing material; and unless there be that solid foundation the nurse of trimmings and accomplishments is not a satisfactory article for use in the sick room. To recall a few practical points, it was not an uncommon experience in my probationary and nursing days during the hot months for the afternoon milk to reach my wards in a semi-turned condition. Such milk was not suited to the sick, especially for those in a medical ward, yet it was often not sufficiently "turned" to be condemned by the catering authorities. "Someone had blundered," either in the original supply, or it had been received in dairy or hospital in vessels insufficiently scoured, or kept in too hot a place. Now it is certain that no more useful branch of hospital work could be served than the overseeing of the milk so as to prevent *contretemps* such as these.

I have visited the isolation cow-sheds attached to some insane asylums, and have watched the bacteriological examination of the milk undertaken, lest tuberculosis might be present in the institutional supply. Now, without asking for so elaborate and expensive a system as isolated cows and sheds and bacteriological milk examinations for our hospitals, there should be such supervision that the milk sent up for use in the wards should be above suspicion of sourness. Hospital bread, also, should be strictly and hygienically made, and any shortcomings apparent in the butter or other articles of food are practical points to which housewifely attention should be paid. A hundred similar little avenues for the energies of the hospital housekeeper will suggest themselves, and will suggest at the same time the lines on which a definite, practical teaching might tend.

It would be most instructive and satisfactory if my sister-matrons would express their views as to the need existing for some extended teaching in our training schools of hospital housewifery. The cleaning and polishing of the hospital wards has reached a fine art, and it is high time that the dietetics, the cuisine, and the kitchen departments generally of our hospitals were brought to the same pitch of perfection. It requires only a little enterprise and some patience and perseverance to establish a recognition of the fact that we are turning out at the present time a race of trained nurses possessing only the most elementary and superficial knowledge of institutional and invalid housekeeping and the culinary and domestic arts generally. And there can be little doubt that these branches are all-important to the welfare of hospital patients, as well as to the wellbeing of private patients in every grade and station.

